# Sampling Key Populations for HIV Surveillance: Results From Eight Cross-Sectional Studies Using Respondent-Driven Sampling and Venue-Based Snowball Sampling

**DOI:** 10.2196/publichealth.8116

**Published:** 2017-10-20

**Authors:** Amrita Rao, Shauna Stahlman, James Hargreaves, Sharon Weir, Jessie Edwards, Brian Rice, Duncan Kochelani, Mpumelelo Mavimbela, Stefan Baral

**Affiliations:** ^1^ Department of Epidemiology Johns Hopkins Bloomberg School of Public Health Baltimore, MD United States; ^2^ Measurement and Surveillance of HIV Epidemics Consortium Department of Social and Environmental Health Research London School of Hygiene & Tropical Medicine London United Kingdom; ^3^ Department of Epidemiology University of North Carolina Gillings School of Global Public Health Chapel Hill, NC United States; ^4^ Center for Communication Programs Johns Hopkins University Mbabane Swaziland; ^5^ Swaziland National AIDS Program Mbabane Swaziland

**Keywords:** HIV, public health surveillance, health surveys, sex work, sexual minorities, homosexuality, male, Cameroon, Swaziland

## Abstract

**Background:**

In using regularly collected or existing surveillance data to characterize engagement in human immunodeficiency virus (HIV) services among marginalized populations, differences in sampling methods may produce different pictures of the target population and may therefore result in different priorities for response.

**Objective:**

The objective of this study was to use existing data to evaluate the sample distribution of eight studies of female sex workers (FSW) and men who have sex with men (MSM), who were recruited using different sampling approaches in two locations within Sub-Saharan Africa: Manzini, Swaziland and Yaoundé, Cameroon.

**Methods:**

MSM and FSW participants were recruited using either respondent-driven sampling (RDS) or venue-based snowball sampling. Recruitment took place between 2011 and 2016. Participants at each study site were administered a face-to-face survey to assess sociodemographics, along with the prevalence of self-reported HIV status, frequency of HIV testing, stigma, and other HIV-related characteristics. Crude and RDS-adjusted prevalence estimates were calculated. Crude prevalence estimates from the venue-based snowball samples were compared with the overlap of the RDS-adjusted prevalence estimates, between both FSW and MSM in Cameroon and Swaziland.

**Results:**

RDS samples tended to be younger (MSM aged 18-21 years in Swaziland: 47.6% [139/310] in RDS vs 24.3% [42/173] in Snowball, in Cameroon: 47.9% [99/306] in RDS vs 20.1% [52/259] in Snowball; FSW aged 18-21 years in Swaziland 42.5% [82/325] in RDS vs 8.0% [20/249] in Snowball; in Cameroon 15.6% [75/576] in RDS vs 8.1% [25/306] in Snowball). They were less educated (MSM: primary school completed or less in Swaziland 42.6% [109/310] in RDS vs 4.0% [7/173] in Snowball, in Cameroon 46.2% [138/306] in RDS vs 14.3% [37/259] in Snowball; FSW: primary school completed or less in Swaziland 86.6% [281/325] in RDS vs 23.9% [59/247] in Snowball, in Cameroon 87.4% [520/576] in RDS vs 77.5% [238/307] in Snowball) than the snowball samples. In addition, RDS samples indicated lower exposure to HIV prevention information, less knowledge about HIV prevention, limited access to HIV prevention tools such as condoms, and less-reported frequency of sexually transmitted infections (STI) and HIV testing as compared with the venue-based samples. Findings pertaining to the level of disclosure of sexual practices and sexual practice–related stigma were mixed.

**Conclusions:**

Samples generated by RDS and venue-based snowball sampling produced significantly different prevalence estimates of several important characteristics. These findings are tempered by limitations to the application of both approaches in practice. Ultimately, these findings provide further context for understanding existing surveillance data and how differences in methods of sampling can influence both the type of individuals captured and whether or not these individuals are representative of the larger target population. These data highlight the need to consider how program coverage estimates of marginalized populations are determined when characterizing the level of unmet need.

## Introduction

### Background

Although probability sampling is often preferred over nonprobability sampling for ensuring representativeness, there is no gold standard method for measuring the coverage of health services for marginalized populations and, consequently, the magnitude of the gaps in service coverage. This gap in characterization of health service needs is true for a number of different health conditions, including, especially, in the realm of human immunodeficiency virus (HIV). HIV key populations include gay men and other men who have sex with men (MSM), female sex workers (FSW), people who inject drugs, and transgender people. Despite much recent progress that has been made toward reducing the incidence of HIV globally, these key populations continue to bear a significant proportion of the HIV burden across high-, middle-, and low-income countries [1-9]. In a wide range of settings, these same populations have been shown to have suboptimal engagement in the HIV care cascade far below the 90-90-90 goals established by the Joint United Nations Program on HIV/AIDS for 2020 [10]. The stigma affecting these key populations, including stigma from health care workers, friends and family, and community members, makes relying on traditional passive biological and behavioral surveillance methods, such as population-based surveys and sexually transmitted disease (STD) or health clinic surveillance, challenging. Stigma may cause the members of key populations to hide their membership and not want to disclose to others their key population defining behavior. This is especially the case in Sub-Saharan African regions where sex work and sex between men is, generally, criminalized, and the HIV epidemic is often widespread. In addition to the issue of stigma, the lack of a traditional sampling frame for key population groups also makes relying on traditional passive surveillance methods difficult [4].

To get a sense of the disproportionate burden of HIV faced by these key populations, the adult prevalence (aged 15-49 years) of HIV, in Cameroon, for example, is 4.5%, with an estimated prevalence of 37% among MSM according to a respondent-driven sampling (RDS) study, and 24% among FSW in a meta-analysis [11-13]. In Swaziland, a nation experiencing a broadly generalized HIV epidemic with around 26% of people aged 15 to 49 years living with HIV, prevalence is about 13% in MSM and approximately 61% among FSW according to RDS studies [14,15]. In Swaziland and Cameroon, both sex work and sex between men are criminalized, with punishment ranging from fines to imprisonment [16-18].

Whereas key populations remain hard to sample in many settings, multiple recruitment strategies have been developed to improve sampling as a means of characterizing current gaps in service coverage [19]. These approaches can be broadly characterized as those that either leverage networks of key populations or those that focus on geographic hotspots—where groups of key populations congregate, such as brothels, gay clubs, or venues where HIV prevention, treatment, and care services are available [20,21]. Specifically, these approaches have often been used to assess the burden of HIV and other sexually transmitted infections (STI), as well as the current coverage of evidence-based services. Although there remains no universal standard for sampling key populations, the implications of using a particular sampling approach appear to be significant [22]. The results often are used to define the current coverage of services and, in turn, the necessary investment for HIV prevention, care, and treatment services for key populations [23-25]. Many studies seek to enroll generalizable samples of MSM and FSW to measure important indicators related to the HIV epidemic and to inform public health programs. However, concerns about insufficient data quality may delay or impede resource allocation decisions [26].

Two of the most commonly used strategies for sampling hard-to-reach populations are snowball sampling and respondent-driven sampling (RDS) [21,27]. Snowball sampling is a process in which seeds are utilized to recruit peers, although the relationship between recruiters and recruits and personal network size are not documented. In snowball sampling, the number of waves reached and the number of coupons issued per recruit is not considered, and the number of seeds can be as high as needed. In addition, sampling biases cannot be adjusted for. The final sample composition is often largely influenced by the initial nonrandom choice of seeds, which also tend to bias participation toward those who are a part of larger social networks [28,29]. Similarly, RDS uses initial recruits or *seeds* to recruit peers through waves of recruitment and allows for calculation of selection probabilities [28-30]. In RDS, the relationship between recruiters and recruits is documented so that recruitment biases can be adjusted and assessed in the analysis [28,29,31,32]. In addition, RDS captures the social network sizes of individuals, so that these can also be taken into account and adjusted for in the analyses [28,29,31,32]. Given the time, resources, and other practical constraints, RDS and snowball sampling can also be used in conjunction with geographical approaches as an alternative to traditional probability and nonprobability sampling strategies by targeting the sampling of key populations at hotspot venues [20,21]. In this study, two of our samples utilized venue-based snowball sampling in which key informants, who worked at community organizations, establishments, clinics, and programs serving MSM or FSW, identified the venues. Study staff visited MSM and FSW venues, and participants were approached at each of the venues to provide an informed written consent and complete the interviewer-administered survey. In many locations, venue managers or key community leaders acted as key informants and assisted the staff in identifying MSM and FSW and facilitating introductions.

### Objective

In this study, we aimed to analyze and compare existing data collected from FSW and MSM using RDS and venue-based snowball sampling approaches in two locations within Sub-Saharan Africa: Manzini, Swaziland and Yaoundé, Cameroon. We compared these samples by a number of characteristics to determine the impact of sampling methodology on inference about these populations. In particular, we examined the prevalence of sociodemographics, self-reported HIV status, frequency of HIV testing, level of experienced stigma, and other HIV-related characteristics. In comparing these characteristics across different samples, we hoped to gain a greater understanding of how sampling methodology influences the type of individuals captured and whether differences in sampling methodology result in differences in underlying target population being measured. Due to the ability of RDS to generate large numbers of recruitment waves [33], we hypothesized that the RDS studies would generate samples with a larger range of HIV prevention and treatment needs than those samples recruited through venue-based snowball samples.

## Methods

### Data Collection and Key Measures

These analyses represent post hoc analyses of data collected by the study investigators. Data were initially collected for HIV bio-behavioral surveillance and population size estimation of MSM and FSW populations in multiple cities in Swaziland [33-39] and Cameroon [40,41]. Manzini and Yaoundé were selected for analyses because these were the only cities in which data were collected for both MSM and FSW using both RDS and venue-based snowball sampling. A summary of the data collection process and key measures is provided below, with more detailed descriptions of study methods provided in Table 1.

#### Swaziland

In Manzini, Swaziland, we recruited 326 MSM and 325 FSW participants using RDS between July and December 2011. A total of 173 MSM and 249 FSW were recruited through snowball sampling through outreach at *hotspot* venues in and near Manzini between October and November 2014. The venues were determined by key informant interviews and then verified by study staff. Outreach at those venues, including referrals, served as the basis for snowball sampling. During all study visits, participants were administered a structured survey instrument that included measures of sociodemographics, self-identified sexual orientation (for MSM), sexual behavior–related stigma, disclosure of sexual practices to family members and health care providers, access to relevant HIV prevention information, HIV-related knowledge, ease in accessing condoms, sexual risk behaviors, and HIV/STI testing and diagnoses [42].

We assessed participants’ level of sexual behavior–related stigma by asking them whether they had ever felt excluded from family gatherings or were afraid to seek health care because of same-sex practices or selling sex, for MSM and FSW, respectively. Disclosure of sexual practices was measured by asking participants whether they had ever told any member of their family or any health care worker that they have sex with other men or that they sell sex. Access to HIV prevention information was measured by asking MSM participants whether they had received any HIV prevention information on sex between men in the past 12 months. Among FSW, this was measured by asking whether participants received any HIV prevention information in the past 12 months. HIV-related knowledge was measured by asking all participants what type of sex carries the most sexual risk for HIV, and what type of lubricant is safest to use for vaginal or anal sex. In addition, participants were asked how difficult or easy it was to access condoms when they need them. Response options were dichotomized to “some access” or “easy access” and “difficult” or “little access.” Network size was captured through self-report. Question-and-answer responses were phrased in the same way across recruitment strategies and for both populations.

For MSM sexual risk behaviors, we assessed the participant’s reported number of male anal sex partners within the past 12 months and whether they had any condomless anal sex in the past 12 months. For FSW, we asked the participants to report their average number of clients per week. There were no comparable condom-use questions for FSW across the RDS and snowball studies. All participants were asked to report whether they had been tested for HIV or STI in the past 12 months. In addition, all participants were asked if they had ever been told by a doctor or health care provider that they have HIV.

#### Cameroon

In Yaoundé, Cameroon, 306 MSM were recruited through RDS from November 2015 to January 2016 and 576 FSW from December 2015 to February 2016. The snowball sampling studies accrued 259 MSM and 308 FSW at venues frequented by these groups between April and May 2013. Similar to Swaziland, venues were determined through key informant interviews and then leveraged as a basis for snowball sampling of key populations.

Similar to the Swaziland studies, all participants in Cameroon were administered a questionnaire that included questions about sociodemographics, self-identified sexual orientation (for MSM), sexual behavior–related stigma, disclosure of sex work to health care providers (FSW), whether any family members knew that they had sex with men (for MSM), and HIV/STI testing and diagnoses. Network size was captured through self-report. Question-and-answer responses, again, were phrased in the same way across recruitment strategy and for both populations. However, information regarding access to relevant HIV prevention information, HIV-related knowledge, ease in accessing condoms, and sexual risk behaviors were either not available or not comparable across recruitment studies and were not included in these analyses.

**Table 1:** Detailed description of study methods.

Study population (recruitment dates)			Detailed sampling methods^a^	Eligibility criteria^b^
**Respondent-driven sampling^c^**			Seeds were recruited from local MSM- or FSW-affiliated communicated-based organizations (although not all seeds were members. Each participant was given three coupons to distribute. Sample size calculations for the initial data collection were powered based on the ability to estimate local HIV prevalence of MSM and FSW, and the required sample sizes were achieved.	Possess valid recruitment coupon
	**Swaziland^d^** **(July - December 2011)**			
		MSM	4 of 5 seeds successfully accrued 326 men through up to 14 waves. A few participants (n=16) could not be traded back to valid sees and were excluded post-hoc.	Report receptive or insertive anal sex with another man for the last 12 months
		FSW	10 of 14 seeds successfully recruited 325 FSW through up to 7 waves	Report exchanging sex for money, favors, or goods in last 12 months
	**Cameroon^e^**			
		MSM (November 2015 - January 2016)	2 of 4 MSM seeds successfully accrued 306 men through up to 12 waves	Report receptive or insertive anal sex with another man for the last 12 months
		FSW (December 2015 - February 2016)	2 of 3 seeds successfully recruited 576 FSW through up to 22 waves	Report more than half income in the past 12 months from sex work
**Snowball sampling**			Participants were recruited through snowball sampling at “hotspot” venues. MSM and FSW venues were visited by study staff, and participants were approached at each of the venues to provide informed written consent and completed the interviewer-administered survey. In many locations, venue managers or key community leaders acted as key informants and assisted staff in identifying MSM and FSW and facilitating introductions.	
	**Swaziland^d^** **(October - November 2014)**		A modified version of the PLACE method [43] was used to characterized venues where MSM and FSW meet new potential sexual partners.	
		MSM	173 MSM were recruited. 7 unique venues in Manzini/Matsapha were mapped and 2 were sampled from.	Report receptive or insertive anal sex with another man for the last 12 months
		FSW	249 FSW were recruited. 12 venues were mapped and 2 were sampled from.	Report exchanging sex for money, favors, or goods in last 12 months
	**Cameroon^e^(April - May 2013)**			
		MSM	259 MSM were recruited. 4 unique venues were mapped and 3 were sampled from.	Report receptive or insertive anal sex with another man for the last 12 months
		FSW	308 FSW were recruited. 15 venues were both mapped and sampled from.	Report more than half income in the past 12 months from sex work

^a^All surveys were conducted using face-to-face interviews in private locations.

^b^All participants had to be at least 18 years of age.

^c^Recruiters were additionally compensated the equivalent of up to US $2 for each participant they recruited into the study.

^d^Participants were reimbursed for their time and travel to the study site dependent on distance traveled to and from the study site.

^e^Participants were reimbursed for the average cost of a meal and transportation (~US $5).

### Ethics

Data collection was approved by the Swaziland Ministry of Health and Scientific Ethics and the Cameroon National Ethics Committee. All studies were also approved by the institutional review board of the Johns Hopkins School of Public Health.

### Statistical Analysis

Crude prevalence estimates were generated for each sample; additionally, 95% CIs were generated for RDS samples. For the RDS samples, RDS-adjusted prevalence estimates (RDS-I estimator) and 95% CIs were created using Respondent-Driven Sampling Analysis Tool (RDSAT Version 7.0, Cornell University, Ithaca, New York). To assess whether or not it was likely that RDS samples had become independent from seed selection, we reported the wave number at which equilibrium was reached. Equilibrium was measured by calculating the cumulative sample proportions of a variable at each wave. When the proportions came within 2% of the final sample proportions at a particular wave, and did not fluctuate more than 2% during the sampling of additional waves, then that wave was considered the *point* of equilibrium [28]. Samples that accrued several waves beyond equilibrium were more likely to be independent from the nonrandom choice of seeds [28]. Crude prevalence estimates from the venue-based snowball samples were compared with the overlap of the RDS-adjusted prevalence estimates between both FSW and MSM in Cameroon and Swaziland.

## Results

### Swaziland

In Manzini, Swaziland, MSM who were recruited using venue-based snowball sampling in 2014, as compared with those recruited through RDS in 2011, tended to be older than 26 years, less likely to belong to the youngest age group (18-21 years), more highly educated, more likely to be employed, less likely to be a student, and less likely to be single/never married (Table 2). In addition, MSM recruited using venue-based snowball sampling were more likely to have received MSM-specific HIV prevention information within the past 12 months, were more likely to report having access or easy access to condoms when they needed them, were less likely to report condomless anal sex in the past 12 months, more likely to report 5 or more sexual partners in the past 12 months, and more likely to have had an STI test in the past 12 months. Venue-based snowball-recruited MSM were also more likely to know what type of sex was riskiest for HIV transmission. Among those self-reporting not living with HIV, MSM recruited using snowball sampling were also more likely to have taken an HIV test in the past 12 months. Finally, MSM recruited using snowball sampling in 2014 were less likely to have disclosed their sexual practices to a health care provider and were less likely to report being afraid to seek health care services.

FSW in Manzini, Swaziland, who were recruited using venue-based snowball sampling in 2014 as compared with RDS in 2011 were older and more highly educated (Table 3). They also had greater levels of HIV prevention knowledge, were more likely to know that anal sex carries the most sexual risk for HIV and that water-based lubricant is the safest type of lubricant to use for vaginal sex. FSW who were recruited using venue-based snowball sampling in 2014 reported greater ease in accessing condoms when they needed them, were more likely to report 10 or more clients per week, were more likely to report having an STI test in the past 12 months, were less likely to self-report being living with HIV, were less likely to have disclosed selling sex to family members, were less likely to feel excluded by family members because of selling sex, were less likely to have disclosed to a health care worker that she sells sex, and were less likely to be afraid of seeking health care services because she sells sex. Among those who self-reported not living with HIV, FSW who were recruited using snowball sampling in 2014 were more likely to report having an HIV test in the past 12 months.

### Cameroon

Among MSM in Yaoundé, Cameroon, compared with those recruited by RDS in 2015-2016, those recruited using venue-based snowball sampling in 2013 tended to be less likely to belong to the youngest (18-21 year) age group, were less likely to have completed only up to a primary school education, were more likely to have completed secondary school/high school, were more likely to be employed, and were less likely to be a student (Table 4). Those recruited using snowball sampling in 2013 were more likely to have received an HIV test in the past 12 months, among those who were not aware of living with HIV. They were also more likely to self-report as not living with HIV and were less likely to self-report being not tested. Finally, those recruited using snowball sampling in 2013 were less likely to report that family members know they have sex with men, were less likely to be treated poorly in a health care center, were more likely to feel like police refuse to protect them, and were more likely to be blackmailed because of having sex with men.

For FSW in Yaoundé, Cameroon, those who were recruited using snowball sampling in 2013 as compared with RDS in 2015-2016 were less likely to belong to the youngest age group (18-21 years), were more likely to be married, were less likely to have completed only a primary school education, and were more likely to have completed secondary school or high school (Table 5). FSW recruited using snowball sampling were more likely to self-report as HIV-negative, were less likely to self-report as HIV-positive, but similarly likely to report having never tested for HIV. Finally, FSW recruited using snowball sampling were more likely to have told a health care worker that she sells sex and were more likely to report feeling like police refused to protect her because she sells sex.

**Table 2:** Crude and respondent-driven sampling (RDS)-adjusted prevalence estimates from men who have sex with men (MSM) recruited using snowball sampling and RDS methods in Manzini, Swaziland.

Characteristics		Snowball	RDS (N=310)		
		n (%)	n (%)	Adjusted % (95% CI)	Equilibrium wave^a^
**Age (years)**					
	18-21	42 (24.3)	139 (44.8)	47.6 (39.2-57.0)	5
	22-25	54 (31.2)	94 (30.3)	29.6 (22.4-36.9)	5
	26+	77 (44.5)	77 (24.8)	22.8 (16.2-30.0)	7
**Education completed**					
	Primary school or lower	7 (4.0)	109 (35.2)	42.6 (33.3-51.4)	4
	Secondary school/high school	88 (50.9)	133 (42.9)	42.4 (34.5-50.5)	9
	More than high school	84 (45.1)	68 (21.9)	15.0 (10.0-21.0)	4
**Employment status**					
	Unemployed	61 (35.3)	96 (32.3)	29.2 (22.3-36.1)	7
	Student	28 (16.2)	101 (34.0)	40.0 (30.1-47.7)	7
	Employed	84 (48.6)	100 (33.7)	30.8 (23.8-41.0)	7
**Single/never married**					2
	Yes	147 (90.7)	295 (96.1)	96.6 (93.0-99.4)	
	No	15 (9.3)	12 (3.9)	3.4 (0.6-7.0)	
**Sexual orientation**					
	Heterosexual	2 (1.2)	5 (1.6)	3.0 (0.1-7.5)	4
	Bisexual	59 (35.1)	112 (36.4)	41.4 (33.6-49.5)	7
	Gay/homosexual	107 (63.7)	191 (62.0)	55.6 (47.3-63.1)	7
**Received HIV prevention info on sex between men, past 12 months**					5
	Yes	122 (73.5)	83 (26.9)	79.3 (74.2-84.8)	
	No	44 (26.5)	226 (73.1)	20.7 (15.2-25.8)	
**Knowledge of what type of sex is riskiest for HIV transmission**					7
	Yes	69 (40.1)	74 (24.0)	20.4 (14.5-27.0)	
	No	103 (59.9)	235 (76.1)	79.6 (73.0-85.5)	
**Knowledge of what type of anal sex is riskiest for HIV transmission**					7
	Yes	51 (29.7)	93 (30.0)	29.2 (23.2-36.4)	
	No	121 (70.4)	217 (70.0)	70.8 (63.6-76.8)	
**Ease in accessing condoms**					5
	Access or easy access	155 (90.1)	249 (80.8)	82.3 (76.2-87.6)	
	Difficult or little access	17 (9.9)	59 (19.2)	17.7 (12.4-23.8)	
**Condomless anal sex, past 12 months**					6
	Yes	56 (34.8)	132 (49.3)	49.3 (39.8-58.4)	
	No	105 (65.2)	136 (50.7)	50.7 (41.6-60.2)	
**Number of male anal sex partners, past 12 months**					
	1	54 (32.5)	143 (46.7)	52.5 (43.8-59.8)	8
	2-4	73 (44.0)	133 (43.5)	41.1 (34.1-49.0)	6
	5 or more	39 (23.5)	30 (9.8)	6.5 (3.3-10.1)	3
**STI test in past 12 months**					3
	Yes	78 (45.3)	40 (13.2)	15.1 (9.8-21.2)	
	No	94 (54.7)	263 (86.8)	84.9 (78.8-90.2)	
**HIV test in past 12 months, among those not living with HIV**					9
	Yes	121 (89.0)	152 (52.9)	52.1 (44.0-60.7)	
	No	15 (11.0)	138 (47.1)	47.9 (39.3-56.0)	
**Self-reported HIV status**					7
	Negative/don’t know/not tested	135 (92.5)	285 (94.4)	94.4 (88.6-97.5)	
	Positive	11 (7.5)	17 (5.6)	5.6 (2.5-11.4)	
**Told family he has sex with men**					4
	Yes	77 (44.5)	166 (53.5)	41.7 (36.1-50.3)	
	No	96 (55.5)	144 (46.5)	58.3 (49.7-63.9)	
**Felt excluded from family because he has sex with men**					4
	Yes	37 (21.4)	80 (25.8)	28.5 (22.0-36.2)	
	No	136 (78.6)	230 (74.2)	71.5 (63.8-78.0)	
**Told health care worker he has sex with men**					5
	Yes	36 (21.3)	94 (30.3)	25.7 (20.4-32.4)	
	No	133 (78.7)	216 (69.7)	74.3 (67.6-79.6)	
**Afraid to seek health care because he has sex with men**					9
	Yes	42 (24.3)	173 (56.4)	60.4 (52.1-67.9)	
	No	131 (75.7)	134 (43.6)	39.6 (32.1-47.9)	

^a^Respondent-driven sampling (RDS) recruitment wave number at which equilibrium was reached for given variable.

**Table 3:** Crude and respondent-driven sampling (RDS)-adjusted prevalence estimates from female sex workers (FSW) recruited using snowball sampling and RDS methods in Manzini, Swaziland.

Characteristics			Snowball		RDS (N=325)				
			n (%)		n (%)		Adjusted % (95% CI)		Equilibrium wave^a^
**Age (years)**									
	18-21	20 (8.0)		82 (25.2)		42.5 (31.6-49.6)		6	
	22-25	63 (25.3)		85 (26.2)		22.9 (20.5-32.1)		4	
	26+	166 (6.7)		158 (48.6)		34.6 (25.4-42.1)		5	
**Education completed**									
	Primary school or lower	59 (23.9)		281 (86.5)		86.6 (78.7-92.3)		3	
	Secondary school/high school	166 (67.2)		40 (12.3)		11.2 (6.9-17.8)		3	
	More than high school	22 (8.9)		4 (1.2)		2.2 (0-5.7)		1	
**Single/never married**									4
	Yes	210 (85.4)		285 (88.8)		89.6 (86.4-95.4)			
	No	36 (14.6)		36 (11.2)		10.4 (4.6-13.6)			
**Received HIV prevention info, past 12 months**									3
	Yes	211 (85.1)		276 (86.0)		84.0 (77.8-91.7)			
	No	37 (14.9)		45 (14.0)		16.0 (8.3-22.2)			
**Knowledge of what type of sex is riskiest for HIV transmission**									2
	Yes	62 (25.1)		34 (10.5)		7.0 (4.4-11.5)			
	No	185 (74.9)		290 (89.5)		93.0 (88.5-95.6)			
**Knowledge that water-based lubricant is safest for vaginal sex**									5
	Yes	73 (29.7)		39 (12.1)		10.4 (4.9-15.9)			
	No	173 (70.3)		284 (87.9)		89.6 (84.1-95.1)			
**Knowledge that water-based lubricant is safest for anal sex**									4
	Yes	26 (10.6)		23 (7.3)		3.3 (1.8-6.2)			
	No	219 (89.4)		293 (92.7)		96.7 (93.8-98.2)			
**Ease in accessing condoms**									4
	Access or easy access	242 (98.0)		266 (83.1)		87.3 (82.3-90.9)			
	Difficult or little access	5 (2.0)		54 (16.9)		12.7 (9.1-17.7)			
**Number of clients per week**									
	0-3	55 (22.5)		95 (29.9)		36.3 (25.9-42.9)		3	
	4-9	104 (42.6)		143 (45.0)		46.4 (39.3-54.9)		5	
	10 or more	85 (34.8)		80 (25.2)		17.3 (12.8-25.6)		4	
**STI test in past 12 months**									4
	Yes	145 (58.7)		89 (27.5)		24.9 (19.4-31.6)			
	No	102 (41.3)		235 (72.5)		75.1 (68.4-80.6)			
**HIV test in past 12 months, among those not living with HIV**									4
	Yes	121 (83.4)		92 (62.6)		55.5 (45.9-80.1)			
	No	24 (16.6)		55 (37.4)		44.5 (19.9-54.1)			
**Self-reported HIV status**									5
	Negative/don’t know/not tested	142 (61.7)		146 (45.3)		44.5 (38.5-53.2)			
	Positive	88 (38.3)		176 (54.7)		55.5 (46.8-61.5)			
**Told family she sells sex**									3
	Yes	45 (18.9)		98 (30.2)		24.8 (19.2-32.0)			
	No	203 (81.9)		226 (69.8)		75.2 (68.0-80.8)			
**Felt excluded from family because she sells sex**									4
	Yes	32 (13.0)		124 (38.3)		37.5 (28.9-43.2)			
	No	215 (87.0)		200 (61.7)		62.5 (56.8-71.1)			
**Told health care worker she sells sex**									5
	Yes	20 (8.1)		84 (25.9)		14.6 (10.4-20.5)			
	No	226 (91.9)		240 (74.1)		85.4 (79.5-89.6)			
**Afraid to seek health care because she sells sex**									4
	Yes	38 (15.3)		143 (44.0)		41.2 (32.9-47.9)			
	No	211 (84.7)		182 (56.0)		58.8 (52.1-67.1)			

^a^Respondent-driven sampling (RDS) recruitment wave number at which equilibrium was reached for given variable.

**Table 4:** Crude and respondent-driven sampling (RDS)-adjusted prevalence estimates from men who have sex with men (MSM) recruited using snowball sampling and RDS methods in Yaoundé, Cameroon.

Characteristics		Snowball	RDS (N=306)		
		n (%)	n (%)	Adjusted % (95% CI)	Equilibrium wave^a^
**Age (years)**					
	18-21	52 (20.1)	99 (32.4)	47.9 (35.7-57.5)	9
	22-25	101 (39.0)	106 (34.6)	32.6 (23.6-43.6)	6
	26+	106 (40.9)	101 (33.0)	19.5 (13.6-27.6)	9
**Education completed**					
	Primary school or lower	37 (14.3)	138 (45.1)	46.2 (35.9-57.4)	9
	Secondary school/high school	112 (43.2)	29 (9.5)	15.3 (6.9-24.8)	8
	More than high school	110 (42.5)	139 (45.4)	38.5 (29.2-48.4)	9
**Employment status**					
	Unemployed	31 (12.5)	51 (16.8)	12.9 (7.4-20.6)	7
	Student	103 (41.5)	159 (52.3)	63.8 (53.3-73.3)	8
	Employed	114 (46.0)	94 (30.9)	23.4 (15.0-32.5)	7
**Married**					4
	Yes	7 (2.7)	9 (2.9)	1.0 (0.3-2.2)	
	No	251 (97.3)	297 (97.1)	99.0 (97.8-99.7)	
**Sexual orientation**					
	Heterosexual	3 (1.2)	1 (0.3)	1.5 (0.0-4.1)	4
	Bisexual	158 (62.0)	187 (61.9)	59.4 (50.1-70.5)	11
	Gay/homosexual	94 (36.9)	114 (37.8)	39.2 (28.2-49.0)	11
**HIV test in past 12 months, among those not living with HIV**					11
	Yes	188 (88.7)	152 (67.6)	55.9 (42.3-73.3)	
	No	24 (11.3)	73 (32.4)	44.1 (26.7-57.7)	
**Self-reported HIV status**					
	Negative	212 (88.3)	174 (59.8)	60.6 (48.8-71.4)	7
	Positive	14 (5.8)	65 (22.3)	15.1 (8.6-24.5)	10
	Not tested	14 (5.8)	52 (17.9)	24.3 (14.2-34.7)	11
**Family knows he has sex with men**					7
	Yes	117 (45.5)	182 (59.5)	57.9 (48.1-67.7)	
	No	140 (54.5)	124 (40.5)	42.1 (32.3-51.9)	
**Treated poorly in health care center because he has sex with men**					6
	Yes	10 (3.9)	27 (8.8)	7.4 (3.6-12.0)	
	No	248 (96.1)	279 (91.2)	92.6 (88.0-96.4)	
**Felt like police refused to protect him because he has sex with men**					9
	Yes	39 (15.1)	26 (8.5)	7.1 (3.6-11.9)	
	No	219 (84.9)	280 (91.5)	92.9 (88.1-96.4)	
**Blackmailed because he has sex with men**					7
	Yes	116 (45.3)	113 (36.9)	32.6 (24.0-42.3)	
	No	140 (54.7)	193 (63.1)	67.4 (57.7-76.0)	

^a^Respondent-driven sampling (RDS) recruitment wave number at which equilibrium was reached for given variable.

**Table 5:** Crude and respondent-driven sampling (RDS)-adjusted prevalence estimates from female sex workers (FSW) recruited using snowball sampling and RDS methods in Yaoundé, Cameroon.

Characteristics		Snowball	RDS (N=576)		
		n (%)	n (%)	Adjusted % (95% CI)	Equilibrium wave^b^
**Age (years)**					
	18-21	25 (8.1)	75 (13.0)	15.6 (10.3-19.0)	12
	22-25	68 (22.1)	129 (22.4)	26.7 (21.8-33.1)	15
	26+	215 (69.8)	372 (64.6)	57.7 (51.2-65.0)	15
**Education completed**					
	Primary school or lower	238 (77.5)	520 (90.3)	87.4 (81.8-92.2)	12
	Secondary school/high school	60 (19.5)	22 (3.8)	4.3 (2.4-7.1)	8
	More than high school	9 (2.9)	34 (5.9)	8.3 (4.0-13.1)	13
**Married**					3
	Yes	19 (6.3)	17 (3.0)	4.5 (1.9-7.6)	
	No	285 (93.8)	559 (97.0)	95.5 (92.4-98.1)	
**HIV test in past 12 months, among those not living with HIV**					14
	Yes	182 (70.3)	291 (70.0)	65.2 (55.7-72.1)	
	No	77 (29.7)	125 (30.1)	34.8 (27.9-44.3)	
**Self-reported HIV status**					
	Negative	254 (83.3)	434 (76.3)	76.8 (71.7-81.9)	8
	Positive	21 (6.9)	75 (13.2)	10.5 (6.5-14.7)	9
	Not tested	30 (9.8)	60 (10.5)	12.7 (8.9-16.7)	10
**Told a health care worker that she sells sex**					14
	Yes	107 (34.7)	85 (14.8)	15.9 (12.1-21.7)	
	No	201 (65.3)	491 (85.2)	84.1 (78.3-87.9)	
**Treated poorly in health care center because she sells sex**					6
	Yes	5 (1.6)	3 (0.5)	0.1 (0.0-0.3)	
	No	300 (98.4)	573 (99.5)	99.9 (99.7-1.0)	
**Felt like police refused to protect her because she sells sex**					13
	Yes	139 (45.3)	87 (15.1)	13.2 (10.2-17.7)	
	No	168 (54.7)	489 (84.9)	86.8 (82.3-89.8)	
**Blackmailed because she sells sex**					9
	Yes	176 (57.9)	305 (53.0)	56.4 (50.7-62.5)	
	No	128 (42.1)	270 (47.0)	43.6 (37.5-49.3)	

^a^Respondent-driven sampling (RDS) recruitment wave number at which equilibrium was reached for given variable.

## Discussion

### Principal Findings

In these secondary analyses, the Swaziland and Cameroon studies that recruited MSM and FSW using RDS and venue-based snowball sampling methods produced samples that had different compositions for key sociodemographic, stigma, and HIV-related characteristics. In particular, the RDS as compared with the venue-based snowball samples were younger, less educated, had reduced exposure to HIV prevention interventions, had limited access to HIV prevention tools such as condoms, and showed less-reported frequency of STI and HIV testing. MSM and FSW in Swaziland recruited using venue-based snowball sampling as compared with RDS reported larger numbers of sexual partners and sex work clients, respectively, suggesting larger sexual and personal network sizes for individuals in these samples.

In the context of health and HIV surveillance, our snowball sampling studies in Cameroon and Swaziland leveraging *hotspot* venues estimated much higher coverage of services, such as HIV and STI testing and access to condoms, compared with the studies conducted using RDS. Despite the fact that the intent of all of the studies was to capture the same underlying target population, the results presented here suggest that the two sampling methodologies might reach different subgroups of this population.

These analyses are based on studies that were previously conducted for the objective of conducting HIV bio-behavioral surveillance of key populations, and the studies similarly had limited time and resources. As a result, there were some limitations to the application of both approaches in practice. For example, with the exception of FSW in Cameroon, only a small number of venues were mapped and sampled from. Of the sampled venues, the majority of participants were recruited from only a single venue (99.4% of MSM in Manzini, 99.2% of FSW in Manzini, 95.8% of MSM in Yaoundé). If a probability sample of individuals had been taken from either a random selection of venues or a set of venues with special attention to diversity of characteristics of venues (eg, large vs small and high-end vs low-end), coverage estimates may have been closer to the true underlying target population. Even then, those sampled at venues, regardless of the number or diversity of characteristics of venues, may not ever provide a representative estimate of all members of the larger target population. In addition, RDS equilibrium was reached late for some variables, suggesting that in some cases RDS may not have achieved the goal of reaching deeper into population networks, and some traits may still have been dependent on seed selection. Thus, our findings may have been different had these studies reached a greater number of venues or penetrated more deeply into social networks. In particular, the results from the venue-based snowball studies are likely very sensitive to whether program services reached the venue from which the vast majority of participants were recruited. Taking into account this caveat, the limited amount of time and resources available, and the similar objectives of each of the studies to conduct HIV bio-behavioral surveillance of MSM and FSW, RDS in this particular context appears to provide a very different estimate of program coverage when compared with venue-based snowball sampling.

As expected, RDS and venue-based snowball sampling differed in the characteristics of key populations recruited, and as expected, the group recruited by convenience sampling was more likely to be linked to a program, as indicated by the fact that the largest differences among samples were seen for services that participants may have received together (HIV prevention materials, improved HIV knowledge, recent HIV and STI testing). It may be especially important when using venue-based sampling to consider the types of venues used for recruitment and whether or not HIV-related services are also offered either at or through these venues. In sampling key populations, it is important to clarify early and often the intended purpose and use of the generated estimates—that is, who really is the target population and who are we really trying to capture with our surveys and surveillance strategies. For example, in the case of MSM, is the purpose of the study or surveillance system to capture all MSM regardless of degree of prevention or treatment need, the most at-risk MSM who are likely hidden and hard-to-reach, or well-connected MSM who already frequent hotspots and facilities that provide HIV services? Without a clear definition of the target population, it will be difficult to even begin to think about a method for sampling that population. Future studies should work to clearly define their target population and to evaluate, given time and cost constraints, which sampling methodology may be the best fit for different definitions of the target population.

As prioritization of resources and targeted interventions requires a representative estimation of service coverage [24], RDS may be a more useful surveillance tool for capturing key populations with varying HIV prevention needs, particularly in settings with limited existing programs and known venues. However, from a program science perspective [44], it may also be useful to conduct studies using alternative sampling methodology every few years to assess the validity of current programs. Discrepant results between samples recruited using different methodology provides some insight into key components that may be missed or exaggerated for program delivery purposes. Other factors to consider for surveillance program implementation include cost efficiency, long-term sustainability, and potential for scale up [45]. Critical analysis of empirical data will be key to integrating evidence-based approaches for surveillance and translating results into effective programs for key populations across different settings.

In a previous analysis, the prevalence of HIV-related characteristics and other sample characteristics were examined across recruitment waves among MSM in Swaziland, Malawi, and Lesotho [33]. Those findings indicated that men who were recruited in later waves of RDS were more likely to have not tested for HIV and to be unaware of living with HIV [33]. It was hypothesized that by reaching higher number of recruitment waves, the composition of the RDS-generated sample shifted toward individuals who were less engaged in existing HIV prevention services. The results presented in the current analyses are supportive of these prior findings, suggesting that RDS can reach MSM with varying HIV prevention needs if sufficient sample sizes and recruitment waves are achieved. However, results from the current analyses were not as consistent across settings among FSW as they were for MSM. For example, FSW recruited using snowball sampling in Swaziland were not more or less likely than FSW recruited using RDS to have received HIV prevention information, and they were less likely to have HIV transmission prevention knowledge. Further, FSW in Cameroon had similar HIV testing histories regardless of how they were recruited. Perhaps this could be partially attributed to more extensive and uniform uptake of and exposure to targeted HIV-related prevention initiatives for sex workers as compared with MSM in the region in recent years [46,47].

In addition, findings pertaining to the relationship of sampling method with level of sexual practice disclosure and sexual practice–related stigma were mixed. This could be because we were limited to using only a limited number of items to assess stigma and disclosure; namely, those items that were used consistently across the different studies. It could be because of structural- or community-level differences in stigma between the two countries and key population groups [16-18]. Both MSM and FSW who were snowball-sampled at venues as compared with RDS-sampled were less likely to have disclosed their sexual practices to family members and health care workers, with the only exception being that snowball-sampled FSW in Cameroon were more likely than RDS-sampled FSW to have disclosed selling sex to health care workers. Although we might expect those with greater levels of disclosure of sexual practices to be more visible at venues and more *visible*, it should be noted that the converse, that those who have not disclosed are therefore less visible, is not necessarily true. With respect to sexual practice–related stigma among the MSM and FSW, snowball-sampled MSM and FSW in Swaziland reported being less afraid to seek health care services than RDS-sampled MSM and FSW. Given the often geographical targeting of programs, the exposure to programs by these populations may have mitigated the fear of seeking services in health care settings. However, MSM and FSW from venues in Cameroon were more likely than their RDS-sampled counterparts to report feeling like police refused to protect them.

### Limitations

There are several limitations of this study to consider. First, comparisons across studies should be done with caution because our comparisons were limited to the subset of variables that were most similar across existing datasets. Our results may not be generalizable outside Swaziland and Cameroon, although findings may be comparable to other settings where stigma affecting key populations is prevalent. The venue-based snowball samples were smaller in terms of sample size, and recruitment was for a shorter period of time compared with RDS. In addition, RDS and venue-based snowball studies were conducted between 2 and 3 years apart, making these interpretations potentially subject to a period effect. If the source populations changed over time, then the differences in the sample compositions could be because of differing source populations as opposed to different sampling methodologies. However, there were no changes in HIV prevention policies or laws about sex work or same-sex practices during this time, making it unlikely that the composition of key populations in these two cities changed dramatically over this time frame. Further, RDS was conducted before snowball sampling in Swaziland but was conducted after snowball sampling in Cameroon. However, we cannot rule out the possibility of uncontrolled time-varying confounders or changes to the population-level prevalence of characteristics of MSM or FSW over time.

### Conclusions

There remains a sustained and often growing burden of HIV between these two key populations [1-9], reinforcing the need for evidence-based public health and HIV surveillance to inform resource allocation. The findings presented here indicate that samples with varying composition of HIV prevention needs and program exposure are generated by different sampling methodologies. Ultimately, these findings provide further context for understanding existing surveillance data and how differences in methods of sampling can influence both the type of individuals captured and whether or not these individuals are representative of the larger target population. These data highlight the need to consider who we really intend to capture when developing program estimates and how these program coverage estimates of marginalized populations are determined when characterizing the level of unmet need.

## Abbreviations

FSW: female sex workers
HIV: human immunodeficiency virus
MSM: men who have sex with men
NIH: National Institutes of Health
STI: sexually transmitted infections
RDS: respondent-driven sampling
