# Authorship Correction: Sampling Key Populations for HIV Surveillance: Results From Eight Cross-Sectional Studies Using Respondent-Driven Sampling and Venue-Based Snowball Sampling

**DOI:** 10.2196/publichealth.9407

**Published:** 2018-01-15

**Authors:** Amrita Rao, Shauna Stahlman, James Hargreaves, Sharon Weir, Jessie Edwards, Brian Rice, Duncan Kochelani, Mpumelelo Mavimbela, Stefan Baral

**Affiliations:** ^1^ Department of Epidemiology Johns Hopkins Bloomberg School of Public Health Baltimore, MD United States; ^2^ Measurement and Surveillance of HIV Epidemics Consortium Department of Social and Environmental Health Research London School of Hygiene & Tropical Medicine London United Kingdom; ^3^ Department of Epidemiology University of North Carolina Gillings School of Global Public Health Chapel Hill, NC United States; ^4^ Center for Communication Programs Johns Hopkins University Mbabane Swaziland; ^5^ Swaziland National AIDS Program Mbabane Swaziland

In the paper by Amrita Rao et al, “Sampling Key Populations for HIV Surveillance: Results from Eight Cross-Sectional Studies using Respondent-Driven Sampling and Venue-Based Snowball Sampling,” a mistake was made in the metadata and Duncan Kochelani was included twice, while Brian Rice was not included. Brian Rice was always an intended author of this publication. The authors have records of previous versions that had been reviewed by the editorial team in which Brian is included as an author, as well as the final publishing agreement signed by all authors, with both Duncan and Brian as coauthors of this work.

The corrected article will appear in the online version of the paper on the JMIR website on January 15, 2018, together with the publication of this correction notice. Because this was made after submission to PubMed or Pubmed Central and other full-text repositories, the corrected article also has been re-submitted to those repositories.

